# Non-phosphorylatable mutants of Ser184 lead to incomplete activation of Bax

**DOI:** 10.3389/fonc.2022.1068994

**Published:** 2023-01-19

**Authors:** Lilit Simonyan, Mathilde Gonin, James Hanks, Jordan Friedlein, Kevin Dutrec, Hubert Arokium, Akandé Rouchidane Eyitayo, Toukounou Megann Doudy, Stéphane Chaignepain, Stéphen Manon, Laurent Dejean

**Affiliations:** ^1^ Université de Bordeaux, Centre National de la Recherche Scientifique (CNRS), Institut de Biochimie et de Génétique Cellulaires (IBGC), Bordeaux, France; ^2^ California State University of Fresno, Department of Chemistry and Biochemistry, Fresno, CA, United States; ^3^ Université de Bordeaux, CNRS, Centre de Génomique Fonctionnelle Bordeaux (CGFB), Bordeaux, France

**Keywords:** apoptosis, bax, BCL-2 family, mitochondria, phosphorylation, conformationnal changes

## Abstract

The S184 residue of Bax is the target of several protein kinases regulating cell fate, including AKT. It is well-established that, *in cellulo*, the substitution of S184 by a non-phosphorylatable residue stimulates both the mitochondrial localization of Bax, cytochrome c release, and apoptosis. However, in *in vitro* experiments, substituted mutants did not exhibit any increase in their binding capacity to isolated mitochondria or liposomes. Despite exhibiting a significant increase of the 6A7 epitope exposure, substituted mutants remain limited in their ability to form large oligomers, suggesting that they high capacity to promote apoptosis in cells was more related to a high content than to an increased ability to form large pores in the outer mitochondrial membranes.

## Introduction

Apoptosis is the major form of programmed cell death in mammals. It is involved in the development and organ morphogenesis, and in tissular homeostasis also. Alterations of apoptosis are responsible for developmental defects and proliferative diseases. Apoptosis is also involved in the response to anti-tumoral therapies, making it one of the most investigated processes of cell biology (1, 2, for reviews).

The intrinsic pathway of apoptosis, often denoted the “mitochondrial pathway”, is activated following intracellular injuries, such as therapies targeting at DNA, and involves the action of Bcl-2 family members on mitochondria. This family is identified as proteins sharing 1 to 4 homology domains (BH1 to BH4) with the anti-apoptotic protein Bcl-2 (3). They are classically classified as multidomain anti-apoptotic proteins (such as Bcl-2, Bcl-xL, Mcl-1, et al), multidomain pro-apoptotic proteins (Bax, Bak, Bok), and BH3-only proteins (Bid, Bim, Puma, et al.) (4).

Bax and Bak form the core of the action of Bcl-2 family members on mitochondria. Both proteins are normally inactive in non-apoptotic cells. Following apoptosis induction, they are activated and are arranged as a large size pore in the outer mitochondrial membrane (OMM) that favors the release of so-called “apoptogenic factors” from the mitochondrial intermembrane space to the cytosol (5, 6). Altogether, these factors contribute to the apoptotic characteristics of the cell through, namely, the activation of caspases.

**Figure 1:**
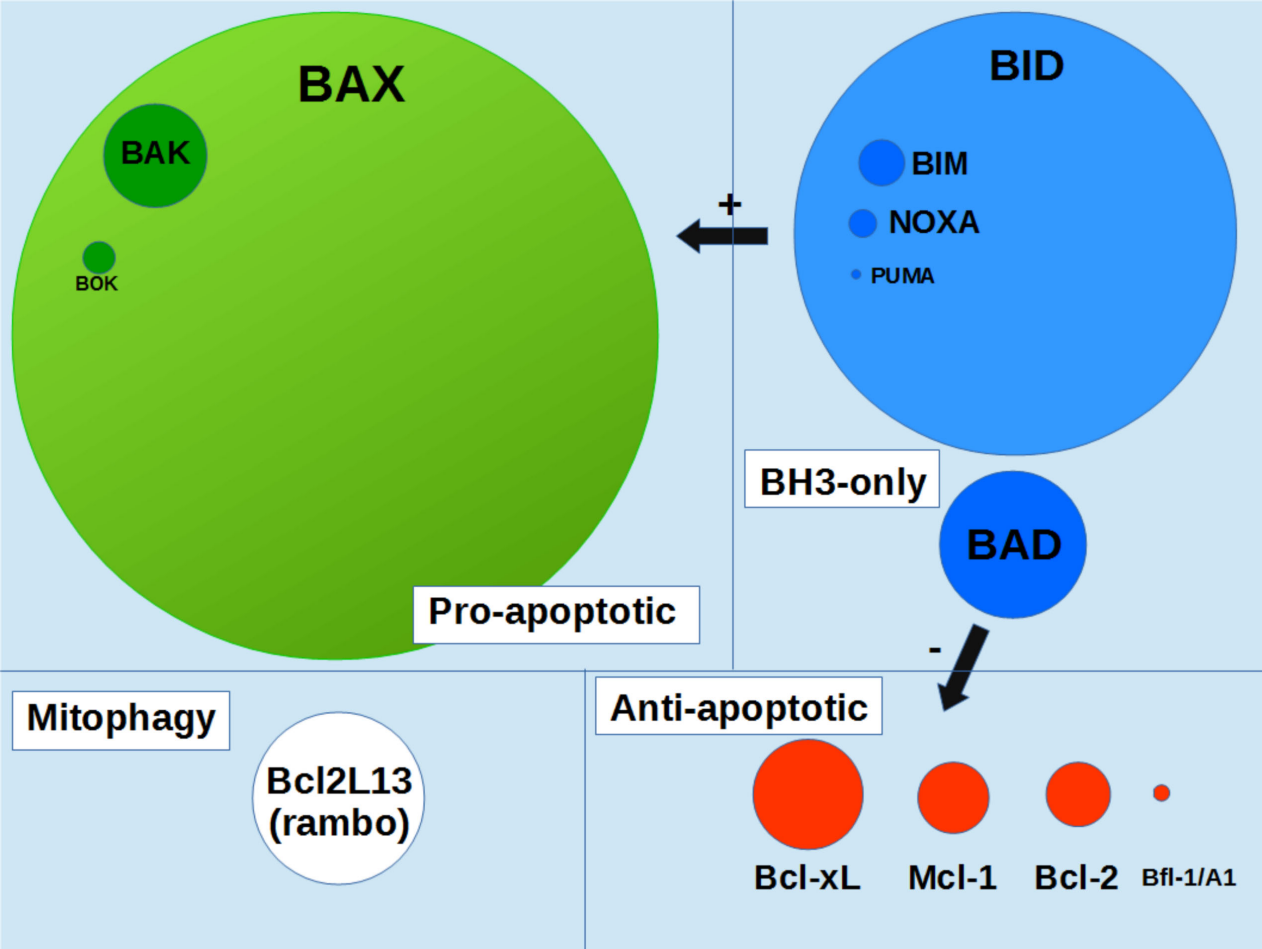
schematic representation of Bcl-2 family proteins content in human organism. Circles area are proportionnal to the protein contents in the whole human organism (integrated values). Data were taken from the Protein Abundance Database (https://pax-db.org). Only the most abundant proteins, for which data are significant, are represented.

Bax is a 21kDa-protein that is expressed at a significant level in most cells. It is by far the most expressed Bcl-2 family member in mammalian tissues (**Figure 1**). The total number of human cells in the human body is estimated to be 3.7 x 10^13^ cells, including 80% of blood cells and the daily turnover of cells is estimated to be around 3.3 x 10^11^ cells (7). 90% of replaced cells are blood cells and 10% are from solid tissues, representing about 0.33 x 10^11^ nonblood cells. This shows that about 0.45% of solid tissues cells are dying (and are replaced) each day, implying that Bax, Bak and Bok should be active in a significant fraction of cells (which can however significantly vary depending on the tissue). It is therefore crucial to understand how Bax function is regulated. Anti-apoptotic proteins Bcl-2 and Bcl-xL are potent inhibitors of Bax function, in the sense that they fully prevent Bax-induced MOM-permeabilization, through the formation of inactive Bax/Bcl-2 (or Bax/Bcl-xL) heterodimers. However, apart from the situation when they are overexpressed (*i.e.* in cancer cells) or in tissues where Bax amount is low (such as in liver), the anti apoptotic proteins are unlikely to contribute significantly to the regulation of Bax, since their relative abundance is significantly much lower (**Figure 1**). Other regulation mechanisms of Bax function, independent from the Bcl-2 family, are therefore likely to exist.

Bax phosphorylation by the survival protein-kinase AKT has been identified early (8). Ser184, located in the C-terminal hydrophobic helix α9, was shown as a target of AKT. It was next shown that the suppression of Bax function through AKT-induced phosphorylation of Ser184 could explain the anti-apoptotic effect of nicotine (9). Other kinases could also phosphorylate Bax (10), and this residue is also a target of phosphatases (11). Ser184 had been previously identified as a key residue in the biochemical properties of helix α9 (12). Indeed, while wild-type full-length Bax does not bind spontaneously to membranes (see also 13), a mutant carrying a deletion of Ser184 (ΔS184) did, suggesting that the deletion favored a movement of α9-helix towards a conformation compatible with membrane insertion (12). As a matter of fact, while a RFP-Hα9 fusion did no bind spontaneously to mitochondria, a RFP-Hα9-ΔS184 fusion did (14). Furthermore, contrary to wild-type Bax, the mitochondrial insertion of the ΔS184 mutant was independent from the presence of Tom22 and stimulated the further insertion of native BaxWT (14). This led to the hypothesis that, once inserted, membrane Bax could serve as a receptor for cytosolic Bax, in an autocatalytic process (15).

Substitution of BaxS184 by negatively charged residues D or E, mimicking the negative charge of phosphoserine, prevents the mitochondrial localization of Bax, not only in mammalian cells (8, 9, 16), but also following heterologous expression in yeast cells (17, 18). However, rather unexpectedly, the expression of BaxS184D in yeast cells led to a much higher level of cytochrome c release than the expression of BaxWT, a phenomenon that has never been observed in mammalian cells. In addition, the mutant BaxS184D was highly sensitive to proteolytic degradation, but was protected by the co-expression of Bcl-xL (18). Also, Bcl-xL appeared to have a greater affinity for BaxS184D than for BaxWT (or a mutant carrying a S184V substitution) (19). We hypothesized that BaxS184D, although mostly cytosolic, was under a conformation able to permeabilize OMM, and that the small fraction located at the mitochondria was sufficient to promote a large release of cytochrome c. This did not take place in mammalian cells, likely because of the presence of anti-apoptotic proteins, namely Bcl-xL, that forces (non-oligomeric) Bax retrotranslocation (20, 21) or possibly other yet unidentified factors inhibiting Bax. Furthermore, it has been shown that Bax-S184E could bind cBid and remained in solution, thus “depleting” BH3-only proteins in cells and leading to some unexpected anti-apoptotic effect (16).

The substitution S184K also prevents Bax mitochondrial localization in yeast cells (17) and is defective to permeabilize liposomes (22). In this case, the introduction of a large positively charged residue in the middle of Hα9 likely breaks its hydrophobic nature, thus fully preventing Bax membrane insertion and its further capacity to form a pore.

Studies have also been done on mutants where S184 is replaced by a non-phosphorylatable and non-charged residue, A or V. These substitutions stimulate the mitochondrial localization of Bax in mammalian cells (8, 9, 16) and in yeast cells (17). This is associated to an increase of the release of cytochrome c. These observations led investigators to identify small molecules having the capacity to prevent S184 phosphorylation, thus activating Bax and stimulating apoptosis, as possible anti-tumorogenic agents (23, 24).

In the present study, we found that, in spite of having a strong mitochondrial localization *in cellulo*, in accordance to previous studies, Bax carrying Ala or Val substitutions of Ser184 does not efficiently bind to isolated mitochondria. However, these mutants displayed a significant exposure of the 6A7 epitope, suggesting that a conformational change of the N-terminus of the protein took place. The Ser184>Val mutant only formed small-size oligomers, likely tetramers, while a constitutively fully active and membrane-inserted mutant (Pro168>Ala) formed large-size oligomers, likely decamers or dodecamers. This shows that the dephosphorylation of Ser184 is not sufficient to promote a fully active conformation of Bax, and that its capacity to permeabilize the MOM *in cellulo* might be rather related to its high mitochondrial residency (25).

## Results

### BaxS184 non-phosphorylatable substitutions induce a greater mitochondria localization in cellulo but no large increase of MOMP

**Figure 2:**
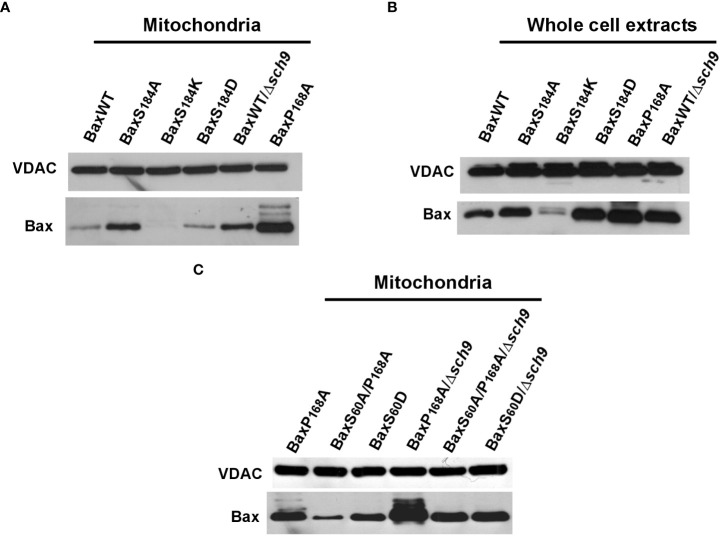
Role of S184 phosphorylation on human Bax mitochondrial localization in yeast. Yeast cells expressing the different Bax mutants were grown until early exponential growth phase on a non-fermentable carbon source. Bax induction was done overnight (~16 hours) and mitochondria were isolated. Western blots were done on 50µg mitochondrial proteins. **(A)** Comparison of the mitochondrial abundance of different Bax mutants substituted on Ser184, in comparison to BaxWT expressed in wild-type W303 or mutant *Δsch9* strains, and to the constitutively mitochondrial and active BaxP168A mutant. **(B)** Western Blots against Bax and VDAC on whole yeast cell extracts, showing that the cellular contents of the different Bax mutants were similar, with the exception of BaxS184K, that was not further included in the study. **(C)** Comparison of the mitochondrial abundance of several active Bax mutants in wild-type W303 and mutant *Δsch9* strains.

**Figure 3:**
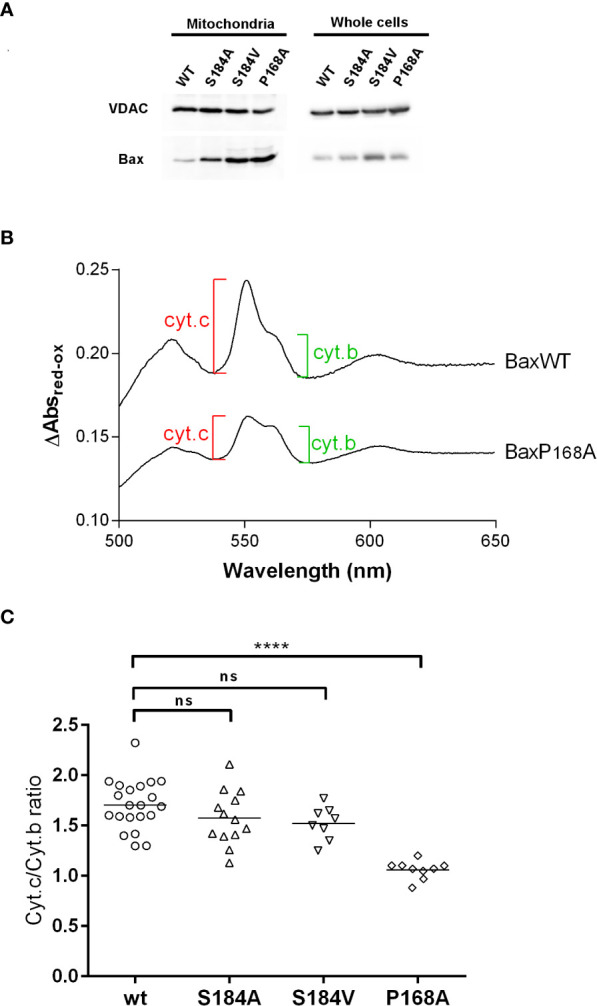
BaxS184V displays a strong mitochondrial localization but a weak capacity to release cytochrome (c) Mitochondria were isolated from yeast cells expressing the indicated Bax mutants. **(A)** Western blots of isolated mitochondria and whole cellular extracts of yeast cells expressing non-phosphorylatable Bax mutants. **(B, C)** Cytochrome c and Cytochrome b contents were measured by redox spectrophotometry on isolated mitochondria. **(B)** Typical redox spectra. A lower Cytochrome c/Cytochrome b ratio indicates a higher Bax capacity to release Cytochrome (c) **(C)** Each point represents a single mitochondria preparation. The horizontal line indicates the mean. Statistical test (unpaired t-test) showed a significant difference between BaxWT and BaxP168A, but not between BaxWT and BaxS184A and BaxS184V. **** means p<0.0001. ns means "not significant".

As reported previously, when heterologously expressed in yeast, the non-phosphorylatable mutant BaxS184A has a greater mitochondrial localization than BaxWT (17, 18) (**Figure 2A**). Conversely, mutants carrying a phosphomimetic (S184D) or a positive charge (S184K) substitution at this position displayed a poor mitochondrial localization. This is in accordance with previous reports showing that the absence of phosphorylation on S184 favored Bax mitochondrial localization in mammalian cells (8, 16). This suggested that a yeast kinase was able to phosphorylate Bax in a similar way as AKT. Interestingly, when BaxWT was expressed in a yeast strain deleted for Sch9, a yeast homolog of AKT (26), BaxWT displayed a greater mitochondrial localization than in a wild-type yeast (**Figure 2A**). We have previously extensively characterized a P168A mutant, that displays both a strong constitutive mitochondrial localization (**Figure 2A**) and a large cytochrome c release capacity (**Figure 3B**) (27–29). The already high mitochondrial localization of this mutant is further increased in a *Δsch9* background (**Figure 2C**) (30), suggesting that the mutation P168A and the absence of phosphorylation on S184 increased Bax mitochondrial localization by distinct mechanisms. Due to the presence of two positively charged residues (KK) in positions 57 and 58, residue S60 of Bax might also be a target of several Ser/Thr kinases (31), and we observed that a phosphomimetic substitution S60D increased Bax mitochondrial localization (as compared to BaxWT) while a non-phosphorylatable substitution S60A combined to the BaxP168A mutation decreased the mitochondrial localization of Bax, as compared to BaxP168A alone (**Figure 2C**). However, like for the BaxP168A mutant, the deletion of Sch9 increased the mitochondrial localization of both BaxS60D and BaxS60A/P168A mutants, showing that Sch9 was not involved in the phosphorylation of S60 and that other yeast kinases might also regulate Bax phosphorylation.

We then asked whether the greater mitochondrial localization of Bax of non phosphorylatable mutants on S184 correlated with a greater Bax activity. Yeast mitochondria were isolated, and the mitochondrial cytochromes content was measured by redox spectrophotometry (**Figure 3B**) (18).

Contrary to BaxP168A, the mutant BaxS184A did not induce a significantly larger cytochrome c release than BaxWT (**Figure 3C**). Since this might be related to the fact that it displayed a lower mitochondrial localization than BaxP168A (**Figures 2A**, **3B**), we constructed another non-phosphorylatable substitution mutant, BaxS184V, that displayed about the same mitochondrial localization as BaxP168A (**Figure 3A**). However, this mutant BaxS184V was only marginally more active than BaxS184A and remained significantly less active than BaxP168A (**Figure 3C**).

### BaxS184 non-phosphorylatable substitutions do not increase mitochondria binding *in vitro*

**Figure 4:**
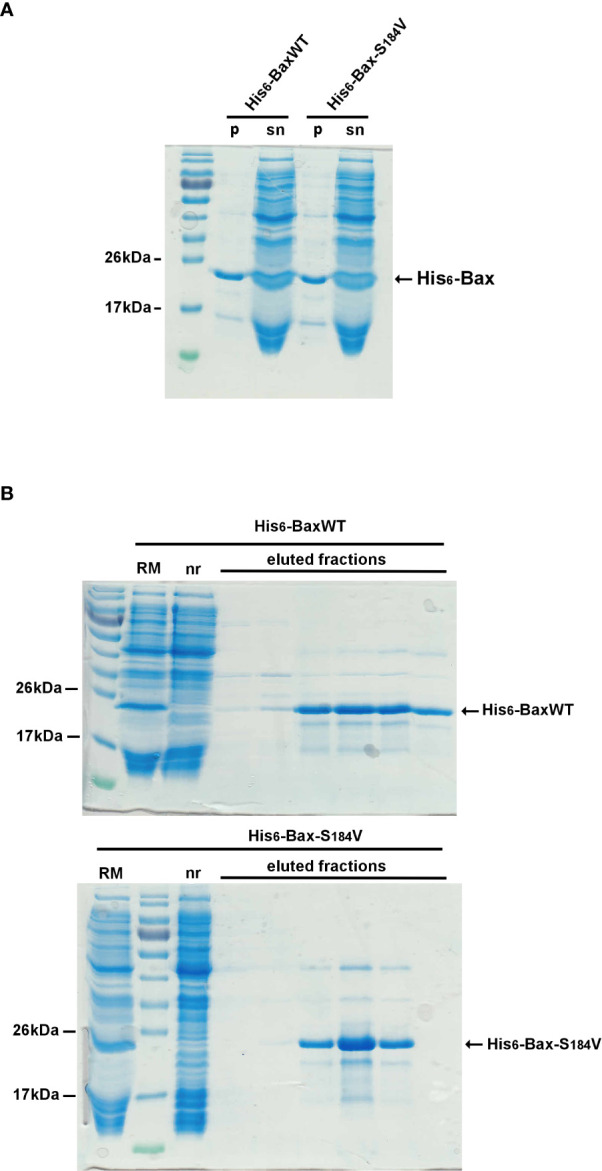
Cell-free production and purification of N-terminal His6-tagged BaxWT and BaxS184V After a 16-hours cell-free protein synthesis (see methods), the reaction mix was centrifuged (10,000 x g, 5 minutes) to eliminate precipitated protein. The vast majority of BaxWT and BaxS184V remain soluble (**Figure 4A**, lanes “sn”) while a small fraction is precipitated (**Figure 4A**, lanes “p”). The supernatants (**Figure 4B**, lanes “RM”) were then loaded on a Ni-NTA FPLC column (His-Trap, Cytiva) through a closed circuit, overnight. Non retained proteins (**Figure 4B**, lanes “nr”) were eliminated through washing with 5 volumes of the loading buffer and 5 volumes of the same buffer supplemented with 20mM imidazole. Retained His6-tagged Bax was eluted with the same buffer containing 250mM imidazole, and further dialyzed to eliminate imidazole. Apart from the initial amount of F8-TAC during the synthesis, no additional detergent or surfactant was added.

To investigate why the mutant BaxS184V was so poorly active, despite being strongly associated to mitochondria, recombinant His_6_-BaxS184V was produced by the same cell-free synthesis method that we have previously used for His_6_-BaxWT and His_6_-BaxP168A (29). The proteins were produced in the presence of a small concentration of the fluorinated surfactant F8-TAC, to maintain the vast majority of the protein (~90%) in solution (**Figure 4B**). Like in previous experiments with BaxWT and BaxP168A (29), no additionnal F8-TAC was added during the purification of His_6_-tagged proteins on His-Trap columns. The small amount of surfactant tightly bound to the proteins was likely sufficient to maintain the solubility of proteins during the purification (**Figure 4B**). Cell-free synthesis of mutants His_6_-BaxS184A and His_6_-BaxS184D gave similar results (not shown).

**Figure 5:**
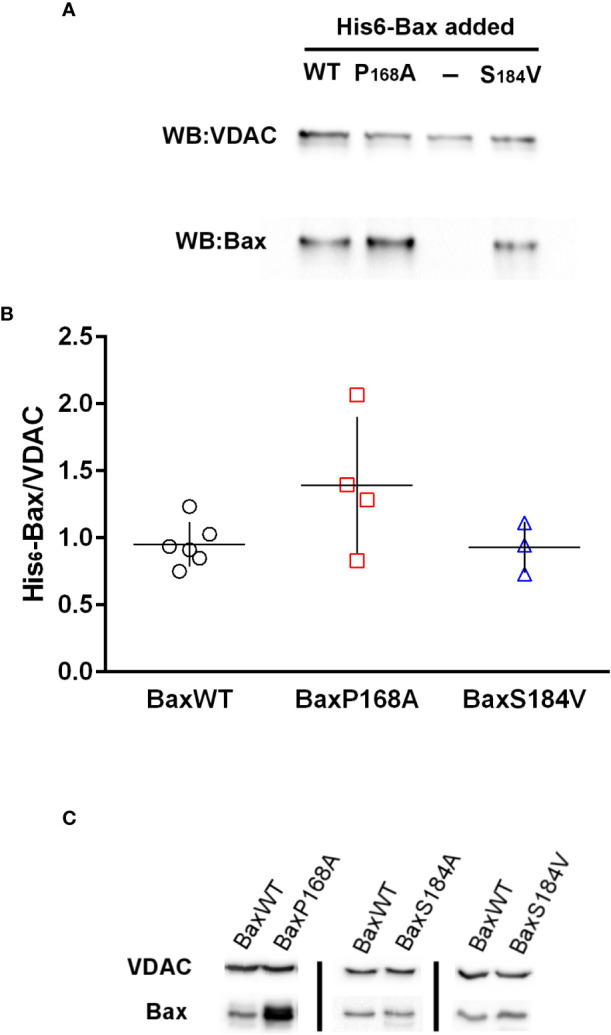
Bax-binding to mitochondria isolated from HCT116 Bax^-/-^ cell line and yeast strain W303 Binding of His6-Bax (WT and mutants) was measured as described in the methods section. **(A)** Representative western-blot of His6-Bax and VDAC (as a loading control) of 50µg of mitochondrial proteins incubated in the presence of different Bax mutants **(B)** Quantification of Bax/VDAC ratios measured on non-saturated western-blots. Each point represents a single mitochondria preparation. The horizontal lines represent the means and vertical lines represent the standard deviation. **(C)** Representative western-blots of His6-Bax and yeast VDAC (as a loading control) of 100µg of yeast mitochondrial proteins incubated in the presence of different Bax mutants.

The binding of recombinant His_6_-Bax mutants on mitochondria isolated from HCT116 Bax-KO cells was measured (**Figures 5A, B**). As previously described (29), the binding of His_6_-BaxP168A appeared to be slightly increased, as compared to the binding of His_6_-BaxWT, although the difference was non statistically significant (p=0.07). It should be noted that the increase of BaxP168A binding to isolated mitochondria compared to BaxWT was much lower than the increase observed when the proteins were expressed in yeast (**Figures 2A**, **3A**; 27) or in mammalian cells (28), showing that other cellular factors, absent from mitochondria preparations, are involved. Opposite to His_6_-BaxP168A, the binding of His_6_-BaxS184V was not increased and remained close to that of His_6_-BaxWT. Again, this is different from *in cellulo* observations, both in yeast (17, 18) and in mammalian cells (8, 9, 16). Similar results were observed with the binding of Bax mutants on isolated yeast mitochondria (**Figure 5C**; 29).

**Figure 6:**
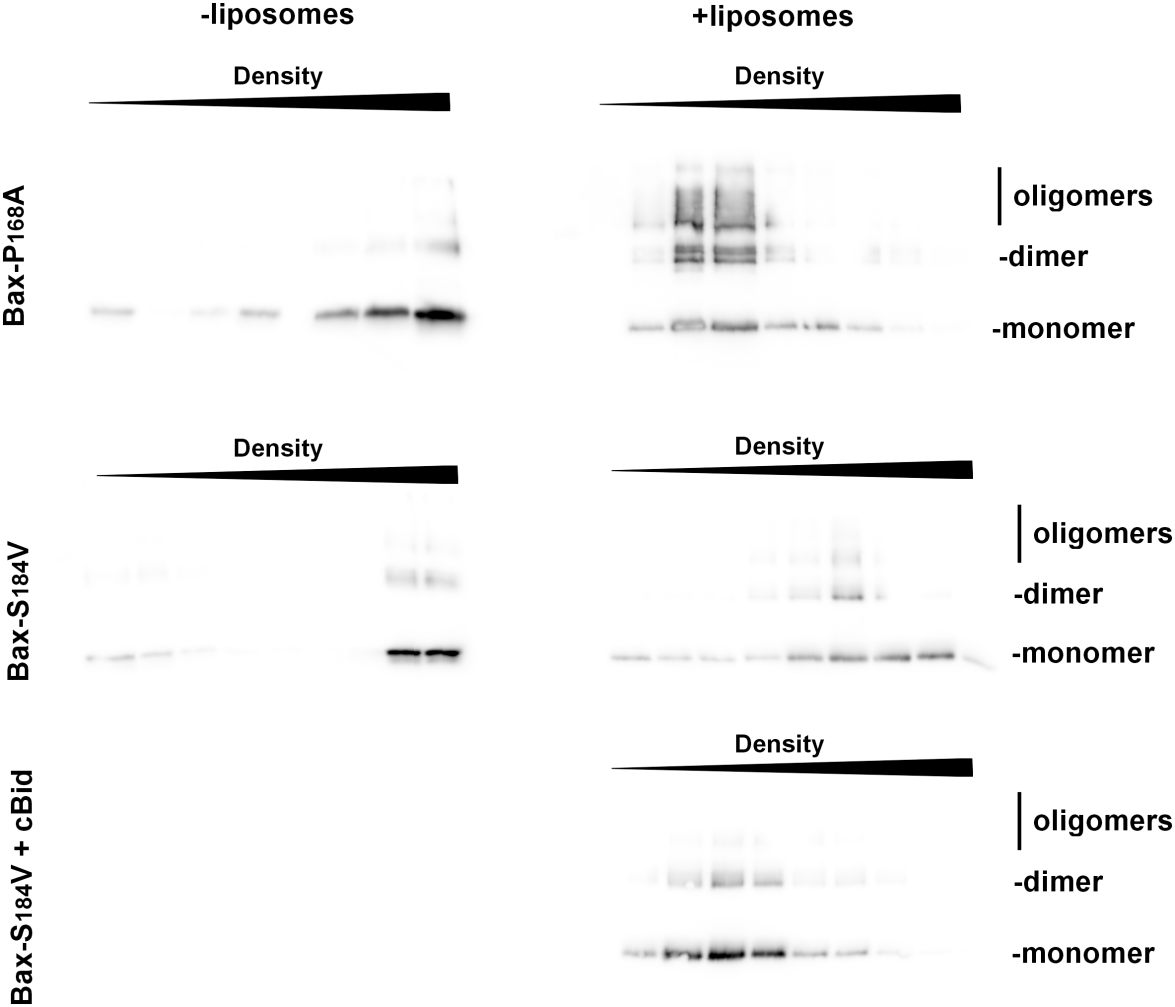
BaxS184V does not spontaneously bind to liposomes, contrary to BaxP168A. Flotation assays of BaxP168A and BaxS184V, mixed or not with liposomes, were done as described in the method section. After the gradients were fractionated, fractions were loaded on SDS-PAGE and revealed by Western-blot. The sizes of monomer (21,5kDa), dimer (43kDa) and oligomers (>65kDa) are indicated. While BaxP168A floated in the presence of liposomes, BaxS184V showing that it is not spontaneously inserted. However, it was inserted when cBid was present.

Similar results were obtained for the binding to liposomes, measured by a flotation assay. While His_6_-BaxP168A spontaneously bound to liposomes, His_6_-BaxS184V did not (**Figure 6**). Also, contrary to His_6_-BaxP168A, His_6_-BaxS184V did not form a significant number of stable oligomers (**Figure 6**). However, as expected, the mutant BaxS184V could still be activated by cBid (**Figure 6**), showing that the mutation did not impair its capacity to sustain conformational changes.

### BaxS184 non-phosphorylatable substitutions induce the exposure of the 6A7 epitope but incomplete Bax oligomerization

**Figure 7:**
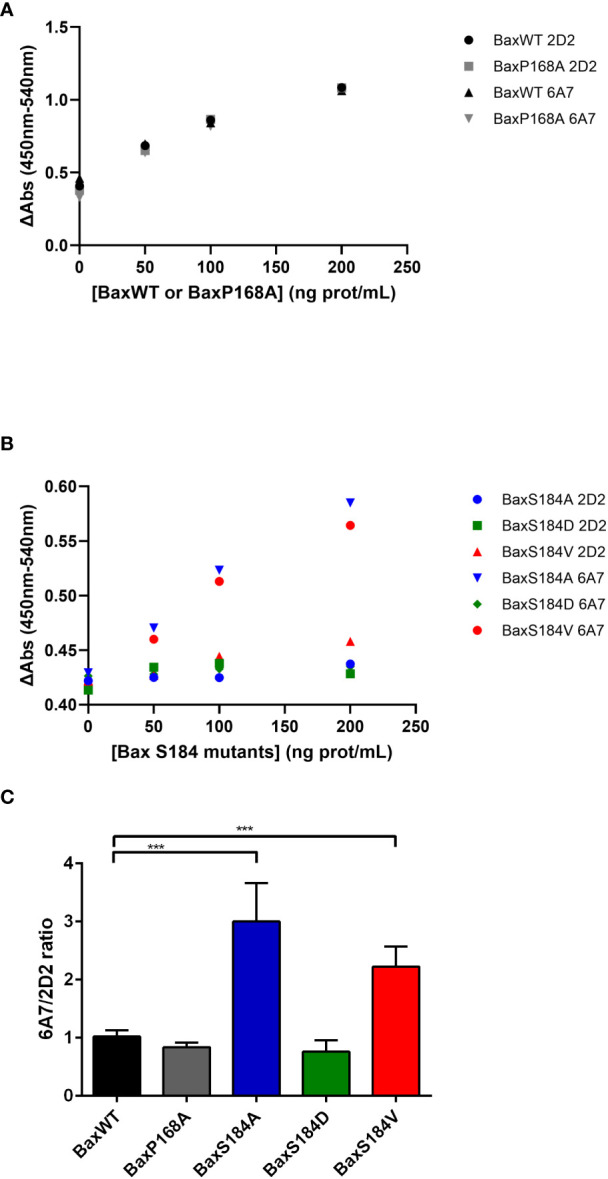
The 6A7 epitope of BaxS184A and BaxS184V is spontaneously exposed. Sandwich Elisa with anti-Bax monoclonal antibodies 2D2 and 6A7 were done on purified Bax mutants as described in the method section. **(A)** Sandwich Elisa performed using 2D2 and 6A7 anti-Bax monoclonal antibodies on BaxWT and BaxP168A (representative experiments) **(B)** Sandwich Elisa performed using 2D2 and 6A7 anti-Bax monoclonal antibodies on BaxS184 mutants (BaxS184A, BaxS184D and BaxS184V) (representative experiments). **(C)** The ratios 6A7/2D2 were calculated for each independent experiment. Student’s impaired t-test were calculated for each mutant in comparison to BaxWT (n= 3 to 6). *** means p<0.001.

The activation process of Bax involves conformational changes of the protein, namely on its N-terminal end. It has been initially shown that several monoclonal antibodies directed against the N-terminal domain of Bax could discriminate between the activated and non-activated conformations of the protein (32). The extent of the recognition by 6A7 (compared to that by the non-discriminating antibody 2D2) has been quantified by an ELISA assay (33). It was observed that the 6A7 antibody binds to the BaxS184A and the BaxS184V mutants with a higher affinity than the 2D2 antibody (**Figures 7B, C**). On the other hand, the extent of binding of both 2D2 and 6A7 antibodies were similar for BaxWT, the phosphomimetic mutant BaxS184D and the constitutively inserted mutant BaxP168A (**Figures 7A–C**). Interestingly, although constitutively membrane-inserted and active, the mutant BaxP168A did not exhibit any increase of 6A7 exposure, as compared to BaxWT.

**Figure 8:**
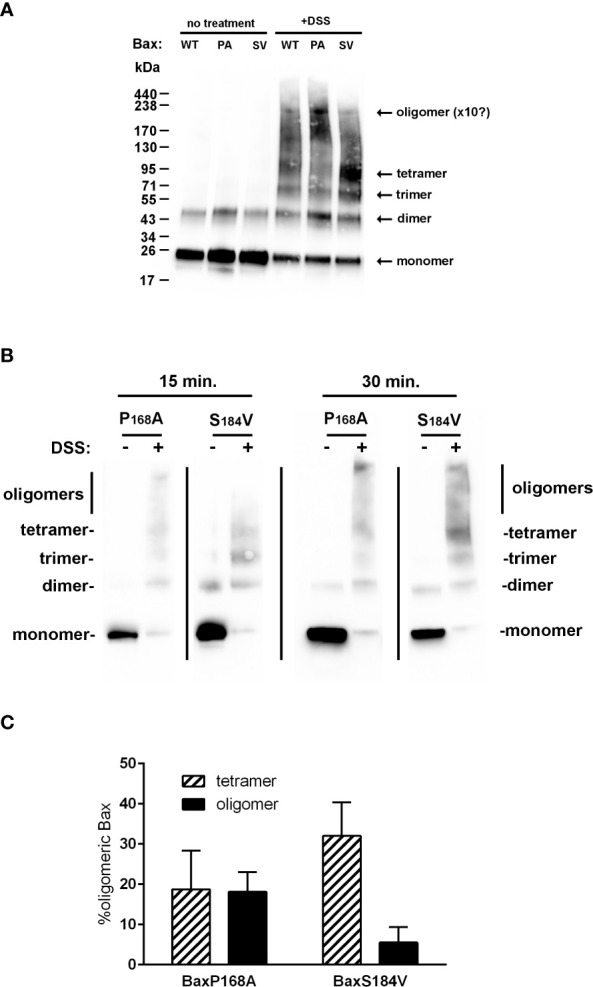
BaxS184V forms oligomers having a smaller size than BaxP168A oligomers. **(A)** Purified Bax mutants were incubated with the amine crosslinking agent DSS for 1 hour and analyzed by western-blot. BaxS184V mostly formed small size oligomers (likely tetramers), while BaxP168A mostly formed large size oligomers (decamers or dodecamers). **(B)** Kinetics of cross-linking of BaxP168A and BaxS184V by DSS showing that, during short incubations, BaxP168A mostly formed large oligomers while BaxS184V mostly formed tetramers, although some oligomers did appear. **(C)** Densitometric quantifications were done on 4 independent experiments for each Bax mutants, and the proportions of tetramers (~85kDa) and high size oligomers (200-250kDa) were calculated.

The interpretation of this observation requires a clear understanding of the meaning of 6A7-recognition. The exposure of the 6A7 epitope does not obligatorily indicate that the full activation of Bax took place (BaxS184A and BaxS184V) and, conversely, Bax oligomerization may occur without any significant exposure of the 6A7 epitope (BaxP168A). The most definitive indication that Bax is fully active is the oligomerization: indeed, it was demonstrated that only Bax oligomers can generate the large pore required to release cytochrome c and other apoptogenic factors (34). We have previously reported that purified recombinant Bax spontaneously auto-assembles with time to form oligomers (29). We then treated BaxWT, BaxP168A and BaxS184V with the crosslinker DSS to stabilize and visualize oligomers (**Figure 8A**). BaxWT formed a range of oligomers from dimers (43kDa) to objects having a size close to 220kDa that might correspond to decamers or dodecamers. BaxP168A formed similar objects and the signal corresponding to the largest objects was stronger than for BaxWT. On the contrary, BaxS184V mostly formed objects that likely corresponded to tetramers, with a size around 85kDa. A comparison of BaxP168A and BaxS184V at two different reaction times showed that, while BaxP168A also formed tetramers, it rapidly accumulated high-size oligomers, while BaxS184V accumulated tetramers and only a small fraction of the protein could form larger size oligomers after a longer reaction time (**Figure 8B**). Densitometric quantifications showed that BaxP168A evenly accumulated both tetramers and high size oligomers, while BaxS184A essentially accumulated tetramers and only a small fraction (~5%) of oligomers (**Figure 8C**).

## Discussion

Data reported in this paper show that the substitution of S184 by a non-phosphorylatable residue A or V only induced a partial activation of Bax. When expressed *in cellulo* (in mammalian cells or in yeast), these non-phosphorylatable mutants displayed a greater mitochondrial localization (16, 18), that is associated to a low rate of retrotranslocation (21, 25). However, when tested *in vitro* on isolated mitochondria or in liposomes, their binding was not significantly greater than the binding of non-activated BaxWT and much weaker than the binding of the constitutively membrane inserted mutant BaxP168A (this study). Their increased capacity to release cytochrome c *in cellulo* might therefore be more related to their high mitochondrial content than to their activation. This is in line with observations showing that a greater mitochondrial localization of Bax, even poorly active, is sufficient to promote the release of cytochrome c and subsequent apoptosis (25).

However, these mutants displayed some degree of activation. BaxS184V was able to form small-size oligomers, likely tetramers (85kDa), that might be able to promote some membrane permeabilization, although less efficiently than the larger oligomers formed by BaxP168A (>220kDa). Besides, both BaxS184V and BaxS184A exhibited a large exposure of the 6A7 epitope, indicating that a large conformational change of the N-terminal end of the protein did occur. In the pioneering study by Hsu and Youle (32), the 6A7 monoclonal antibody was not able to immunoprecipitate Bax interacting with anti-apoptotic proteins Bcl-xL or Bcl-2. Many investigators (including us) have often interpreted this observation as the 6A7 antibody being able to fully recognize active Bax. However, in the same set of experiments, the authors showed that the 6A7 antibody did not recognize heterodimeric Bax (in a complex with Bcl-2 or Bcl-xL) in the presence of Triton-X100 or Nonidet-P40, that have been shown to actually activate Bax (*i.e.* to stimulate its capacity to permeabilize membranes) (35). It is therefore inaccurate to state that “6A7 epitope recognition” equals “every active conformation” of Bax, as also shown by the absence of 6A7 exposure of the constitutively active mutant BaxP168A.

Conversely, the resolution of the structure of the Fab fragment of the 6A7 antibody interacting with the Bax epitope showed that the 6A7 recognized a conformation that strongly differs from the known structure of soluble inactive Bax (12) with a large displacement of this epitope by 9.5Å away from the rest of protein (36). However, as underlined by the authors, this conformational change does not recapitulate the whole process leading to Bax activation, that depends on other domains of the protein. For example, a BaxΨ mutant (in which the whole 6A7 epitope is deleted) binds to mitochondria and can permeabilize the OMM to cytochrome c, that is consistent with the loss of the regulatory “ART” domain (37). However, substitutions in the C-terminal half of the α1-helix (residues 26 and 27) fully prevented both BaxΨ-binding and activity, showing that this part of α1 (that is outside from the 6A7 epitope) is required for Bax activation. It was later shown that this might involve an interaction between Bax and Tom22 (14).

It follows that the recognition of Bax by the 6A7 antibody is an early step in the process of Bax activation (that is observed following the interaction with cBid, for example) but does not recapitulate the whole extent of conformational changes supported by Bax along its activation.

Considering the large recognition of both BaxS184A and BaxS184V, it can be suggested that these substitutions initiate the conformational change leading to the exposure of the 6A7 epitope. However, this does not appear to be sufficient to promote a strong membrane insertion *in vitro*. A possible explanation could be that the substitution S184V induced an alteration leading to the inability to display the fully active conformation. However, in flotation assays, the addition of cBid rendered the protein more able to bind to liposomes. This shows that, although the 6A7 epitope of BaxS184V is exposed, the rest of the protein did not spontaneously display the conformational change that is initiated by cBid, but nevertheless still responded to the activator BH3-only protein and might subsequently be fully activated, as shown by experiments in apoptotic mammalian cells (16).

The spontaneous oligomerization of BaxS184V leads to small size oligomers, likely tetramers, while that of the constitutively full active and membrane inserted mutant BaxP168A leads to objects having a size that likely corresponds to decamers or dodecamers (between 200 and 250kDa). This might explain why, in spite of a strong mitochondrial localization and a large exposure of the 6A7 epitope, the BaxS184V mutant remained poorly active, as compared to the BaxP168A mutant, when heterologously expressed in yeast cells (17 and data herein). It has been shown that membrane inserted Bax spontaneously forms dimers, that further self-assemble into oligomers of different sizes, including tetramers (38). The fact that the S184 substitution limited the formation of larger oligomers, would imply that a correct conformation of Hα9 is needed to form these largest oligomers. It is interesting to note that the P168A substitution, that is expected to change the mobility of Hα9 without modifying its conformation, strongly favored the formation of large oligomers (**Figure 8**), in accordance with its high ability to release cytochrome c in mammalian cells (28), in yeast (27) and in liposomes (29), in spite of the fact that it did not expose the 6A7 epitope (**Figure 7**).

From data reported in this study, the non-phosphorylatable mutants of Bax on the S184 residue represent an intermediate activation state of the protein. The fact that this conformation is highly recognized by the 6A7 antibody previously led to the conclusion that it represents a fully activated form of Bax. However, as we show in the present study, it forms small-size oligomers and, contrary to a fully activated mutant, it is not spontaneously inserted on isolated mitochondria or liposomes. S184 is a target of several protein kinases including AKT (8) and PKCζ (9). This led investigators to design small molecules able to interfere with S184 phosphorylation (23, 24). Such molecules might be useful to maintain a high level of Bax activation in cells where these kinases are overactivated, as it happens in PTEN-deficient tumors. Our data suggest that these molecules, although initiating the activation of Bax (or preventing its inhibition by anti-apoptotic kinases) might not be sufficient and would require additional steps to favor the formation of full-size oligomers, which should be designed to improve the effects of these molecules. Interestingly, it has been shown that the activation status of Bax was influencing the response to BH3-mimetics: indeed, the presence of tBid or Bim modulated their effects, even though their primary target are anti-apoptotic proteins (5). This suggest that a combination of this type of molecules, of which the effect against tumoral cells is now well-established (39, 40) and molecules such as the ones developped to specifically target Bax phosphorylation, could form interesting combinations in anti-tumoral treatments. Similarly, screening approaches identified molecules inducing Bax conformational changes related to those induced by its interaction with Bim and/or tBid (41, 42). Bax (and its closely related functional homolog Bak) are at the core of the pro-apoptotic process. Yet, they have for long been overlooked by pharmacology research, likely because their expression often remains unchanged or poorly changed in cancer cells. Hopefully, current advances in the knowledge of both structural and functional aspects of these proteins are likely to stimulate their identification as trackable targets in cancer and other apoptosis related diseases.

## Materials and methods

### Bax expression in yeast and yeast mitochondria preparation

Wild-type human Bax and the substitution mutants were cloned in the pYES3 plasmid, under the control of the *GAL1/10* promoter and transformed into the wild-type yeast strain W303-1A (*mat a, ade1, his3, leu2, trp1, ura3*) (27). Yeast cells were grown aerobically in YNB medium (0.17% Yeast Nitrogen Base (Difco), 0.1% potassium dihydrogenphosphate, 0.5% ammonium sulfate, 0.2% Drop Mix, 0.01% auxotrophic requirements, 2% DL-lactate as a non-fermentable carbon source, pH 5.2). Subcellular fractionation and mitochondria isolation were done according to previously published methods (27, 43, 44). Mitochondria were resuspended in a 10mM Tris/Maleate buffer (pH 6.8) containing 600mM mannitol, 1 mM EGTA and proteases inhibitors cocktail (Complete, Roche). Cytochrome c release was measured by redox spectrophotometry as described previously (27, 43, 44). The W303-*Δsch9::URA3* strain was a kind gift from Prof. Joris Winderickx (KU Leuven, Belgium).

### HCT116-BaxKO culture and fractionation

HCT116-BaxKO cells were obtained from Dr Bert Vogelstein (Baltimore, USA) and grown in McCoy 5A medium (Gibco) supplemented with 5% fetal calf serum (FCS). Growth medium contained penicillin (100 U/mL) and streptomycin (100 μg/mL). Non-confluent cells were scrapped and resuspended in cold DPBS (Gibco) and washed through a 300 x g, 5 min centrifugation at 4°C. All subsequent manipulations were done on ice. Cells were resuspended in a 10mM Hepes/K buffer (pH 7.5) containing 210 mM mannitol, 70 mM sucrose, 1 mM EDTA and proteases inhibitors cocktail (Complete, Roche) (ME buffer). They were broken through passages in a Dounce tissue grinder tube. Cells disruption was checked under a microscope and was stopped when ~80% of the cells were visually broken. Mitochondria-enriched fraction was recovered through two cycles of differential centrifugation (5 min at 800 × g; 20 min at 20,000 × g), and mitochondria were resuspended in the same buffer. Protein concentrations were determined by the BCA method.

### Cell-free synthesis of recombinant His6-Bax

Cell-free synthesis of His_6_-tagged Bax was described previously (29, 45). His_6_-Bax (wild-type and substitution mutants) were cloned in the piVex 2.3MCS plasmid and expressed in the presence of 0.2mM F8-TAC. F8-TAC was added only in the reaction Mix and was omitted from subsequent buffers. After a 18-hours synthesis, the reaction mix containing Bax was diluted in a 20mM Hepes/K buffer (pH 7.8) containing 150mM NaCl, and loaded on a 5mL-His-Trap column (Cytiva) in a closed circuit, overnight. After loading, the column was connected to an Äkta purifier system, and washed with 5 volumes of the same buffer and 5 volumes of the same buffer supplemented with 20mM imidazole. Bound proteins were eluted with the same buffer containing 250mM imidazole. Bax-containing fractions were pooled and dialyzed against a 25mM Hepes/K buffer (pH 7.4) containing 250mM NaCl and 1mM EDTA (Bax buffer), added with 30% glycerol and stored as working aliquots at -80°C.

### *In vitro* Bax binding to mitochondria and LUV

Yeast or human mitochondria were suspended at 1mg/mL in 200µL of a 10mM Hepes/K buffer (pH 7.4) containing 250mM sucrose, 80mM KCl, 2mM MgOAc, 1mM potassium phosphate, 5mM sodium succinate, 1mM ATP, 0.08mM ADP, proteases inhibitor cocktail (Complete, Roche). His_6_-Bax was added at the optimal concentration of 1µg/mg mitochondria. Mitochondria were then incubated at 30°C for 30 min. Mitochondria were pelleted through a 25,000 x g, 10 min centrifugation. The supernatant was collected, and the pellet was resuspended in 100µL water. Proteins in both supernatant and pellet were precipitated with 0.3M trichloroacetic acid (TCA). Protein pellets were washed with 100µL cold acetone, dried and resolubilized in Laemmli buffer for SDS-PAGE.

Large unilamellar vesicles (LUV) were prepared from a PC/PE/PI/PS/CL (46.5/28.5/9/7/7) (w/w/w/w/w) mixture, as described previously (29, 45). Bax binding to LUV was done as decribed previously (29): 5µg of His_6_-Bax was mixed or not with 100µL LUV in a final volume of 750µL of a 10mM Hepes/K buffer (pH 7.4) containing 200mM KCl, 5mM MgCl_2_, and 0.2mM EDTA (LUV buffer) and incubated for 1 hour at 4°C. The mixture was added with an equal volume of 80% histodenz dissolved in LUV buffer and placed at the bottom of an ultracentrifugation tube. 1.5mL of 30% histodenz dissolved in LUV buffer and 1.5mL of LUV buffer alone were successively added. Histodenz gradients were centrifuged overnight at 109,000 x g. Gradients were then fractionated in 0.5mL fractions. Proteins from each fraction were precipitated and resolubilized as above for SDS-PAGE.

### Bax cross-linking

His_6_-Bax variants were diluted at 48µg/mL in Bax buffer, added with 0.2mM Disuccinimidylsuberate (DSS) and incubated at 25°C for 30 min in a final volume of 45µL. The crosslinking reaction was stopped by adding 50mM ammonium bicarbonate and the sample were solubilized in Laemmli buffer for SDS-PAGE.

### Sandwich ELISA

Sandwich ELISAs on recombinant His6-Bax variants were done according to Teijido et al. (33). Briefly, wells were coated overnight with a capture antibody solution containing 0.25 µg/mL of the monoclonal anti-Bax 6A7 or 2D2 antibody (Sigma) in DPBS at 4°C. Wells were then washed with DPBS, 0.05% Tween-20 (Sigma), and incubated for 1h with 200 µL blocking buffer containing 1% BSA in DPBS, followed by washes with regular DPBS. Recombinant Bax samples (4 increasing concentrations per sample; 1-200 ng/mL) were incubated in the pre-coated wells for 2 hours at room temperature. The plate was then washed DPBS, and each sample incubated for 1 hour with a detection antibody solution containing 0.1 µg/mL of the polyclonal anti-Bax N20 antibody (Santa Cruz) in blocking buffer. After incubation, the plate was washed with DPBS and wells were incubated for 1 hour with 0.12 µg/mL goat anti-rabbit biotinylated third antibody (Abcam) in blocking buffer. Wells were washed with DPBS, and incubated for 30 min with 1X streptavidin-HRP (R&D Systems). Short and long DPBS washes were made; and Bax immunodetection was performed by incubating the samples for 10 min in freshly mixed TMB substrate solution (R&D Systems). The developing reaction was stopped by adding 2N H2SO4, and absorbance was read at 450 nm & 540 nm within 30 minutes after stopping the developing reaction. The ΔAbs (450nm-540nm) and the 6A7/2D2 Delta OD ratio were calculated for each sample to estimate the extent of N-terminus exposure for Bax wild type and Bax mutants tested. Statistical differences were estimated using a student t-test (n=3-6 independent experiment for each mutant tested).

### SDS-PAGE and western-blots

Proteins solubilized in Laemmli buffer were separated on home-made 12.5% Tris/glycine SDS-PAGE or commercial 4-16% gradient Tris/glycine SDS-PAGE (Bio-Rad). Proteins were transferred on PVDF membranes that were saturated with 5% milk in PBS-Tween 20 fot 30 min. Primary antibodies were as follows: rabbit polyclonal anti-human Bax N20 (Santa-Cruz, discontinued) (1/5,000e dilution), mouse monoclonal anti-human Bax 2D2 (Santa-Cruz) (1/5,000e dilution), mouse monoclonal anti-Yeast Porin (Novex) (1/20,000e dilution), mouse monoclonal anti-human Porin (Abcam) (1/10,000e dilution), rabbit monoclonal anti-human Bcl-x (Abcam) (1/10,000 dilution). HRP-coupled goat anti-mouse IgG and goat anti-rabbit IgG secondary antibodies were from Jackson Laboratories (1/10,000e dilution). Primary and secondary antibodies were incubated overnight and 45 min, respectively. Western-Blots were revealed by ECL (Luminata Forte, Millipore) and visualized with a digital camera (G-Box, Syngene) with different acquisition times to avoid signal saturation. Non-saturated signals were quantified with Image J.

## Data availability statement

The original contributions presented in the study are included in the article/supplementary material. Further inquiries can be directed to the corresponding author.

## Author contributions

LS, MG, JH, JF, KD, HA, AE, TD and SC performed and analysed the experiments, and critically read the manuscript. SM and LD integrated data, constructed the figures, and wrote the manuscript. All authors contributed to the article and approved the submitted version.
